# The relationship between sleep duration, cognition and dementia: a Mendelian randomization study

**DOI:** 10.1093/ije/dyz071

**Published:** 2019-05-07

**Authors:** Albert Henry, Michail Katsoulis, Stefano Masi, Ghazaleh Fatemifar, Spiros Denaxas, Dionisio Acosta, Victoria Garfield, Caroline E Dale

**Affiliations:** 1 Institute of Health Informatics, University College London, London, UK; 2 Department of Population Science and Experimental Medicine, Institute of Cardiovascular Science, University College London, London, UK; 3 Department of Clinical and Experimental Medicine, University of Pisa, Pisa, Italy

**Keywords:** Sleep duration, Mendelian randomization, cognition, dementia

## Abstract

**Background:**

Short and long sleep duration have been linked with poorer cognitive outcomes, but it remains unclear whether these associations are causal.

**Methods:**

We conducted the first Mendelian randomization (MR) study with 77 single-nucleotide polymorphisms (SNPs) for sleep duration using individual-participant data from the UK Biobank cohort (*N* = 395 803) and summary statistics from the International Genomics of Alzheimer’s Project (*N* cases/controls = 17 008/37 154) to investigate the potential impact of sleep duration on cognitive outcomes.

**Results:**

Linear MR suggested that each additional hour/day of sleep was associated with 1% [95% confidence interval (CI) = 0–2%; *P *=* *0.008] slower reaction time and 3% more errors in visual-memory test (95% CI = 0–6%; *P *=* *0.05). There was little evidence to support associations of increased sleep duration with decline in visual memory [odds ratio (OR) per additional hour/day of sleep = 1.10 (95% CI = 0.76–1.57); *P *=* *0.62], decline in reaction time [OR = 1.28 (95% CI = 0.49–3.35); *P *=* *0.61], all-cause dementia [OR = 1.19 (95% CI = 0.65–2.19); *P* = 0.57] or Alzheimer’s disease risk [OR = 0.89 (95% CI = 0.67–1.18); *P *=* *0.41]. Non-linear MR suggested that both short and long sleep duration were associated with poorer visual memory (*P* for non-linearity = 3.44e^–9^) and reaction time (*P* for non-linearity = 6.66e^–16^).

**Conclusions:**

Linear increase in sleep duration has a small negative effect on reaction time and visual memory, but the true association might be non-linear, with evidence of associations for both short and long sleep duration. These findings suggest that sleep duration may represent a potential causal pathway for cognition.

**Key Messages**

- Both short and long sleep duration have been linked with poorer cognitive outcomes, but it remains unclear whether these associations are causal.
- We conducted a large linear and non-linear Mendelian randomization (MR) study to investigate the potential causal role of sleep duration on multiple cognitive outcomes.
- Our findings suggest that a linear increase in sleep duration is associated with poorer reaction time and visual memory with small effect size, but there is not enough evidence to support associations with cognitive decline, dementia or Alzheimer’s disease.
- Non-linear MR analysis suggests that the true association might be J-shaped, which could explain the small linear-effect size.
- Sleep duration may represent a potential causal pathway for cognition and thus improving sleep habits within the general population might be useful as a potential therapeutic target to improve cognition.

## Introduction

With population ageing, cognitive decline and dementia have become issues of global importance.^1^ Given that there is currently no effective cure for dementia, identification of modifiable risk factors remains a priority.

In recent decades, numerous observational studies have investigated the association between sleep duration and cognitive performance, but results are conflicting and might be subject to limitations such as residual confounding and over-adjustment of potential mediators.^2^^,^^3^ Reverse causation is also possible, since change in sleep duration might be caused by underlying ill-health,^4^ with growing evidence that accumulation of biomarkers for cognitive impairment could affect sleep quality.^5^

Given the difficulties in implementing large-scale randomized trials involving sleep modification, alternative study design such as Mendelian randomization (MR),^6^ where genetic information is used in an instrumental variable framework, can be used to address some of the limitations of observational studies and estimate causality. Due to the random assortment of genes at conception, MR is less prone to conventional confounding issues with respect to confounders being balanced across genotypes in the population. Reverse causation is also minimized, since cognitive impairment cannot affect individuals’ genotypes.^6^

In this study, we performed large-scale, linear and non-linear MR analyses using individual-level data from 395 803 participants of UK Biobank and summary statistics from the International Genomics of Alzheimer’s Project (IGAP) stage I, which includes 17 008 Alzheimer’s disease (AD) cases and 37 154 controls. We sought to investigate the potential causal role of sleep duration on baseline assessments of visual memory and reaction time, prospective decline in visual memory and reaction time, hospital-diagnosed all-cause dementia and AD.

## Methods

### Study participants

**Figure 1.**
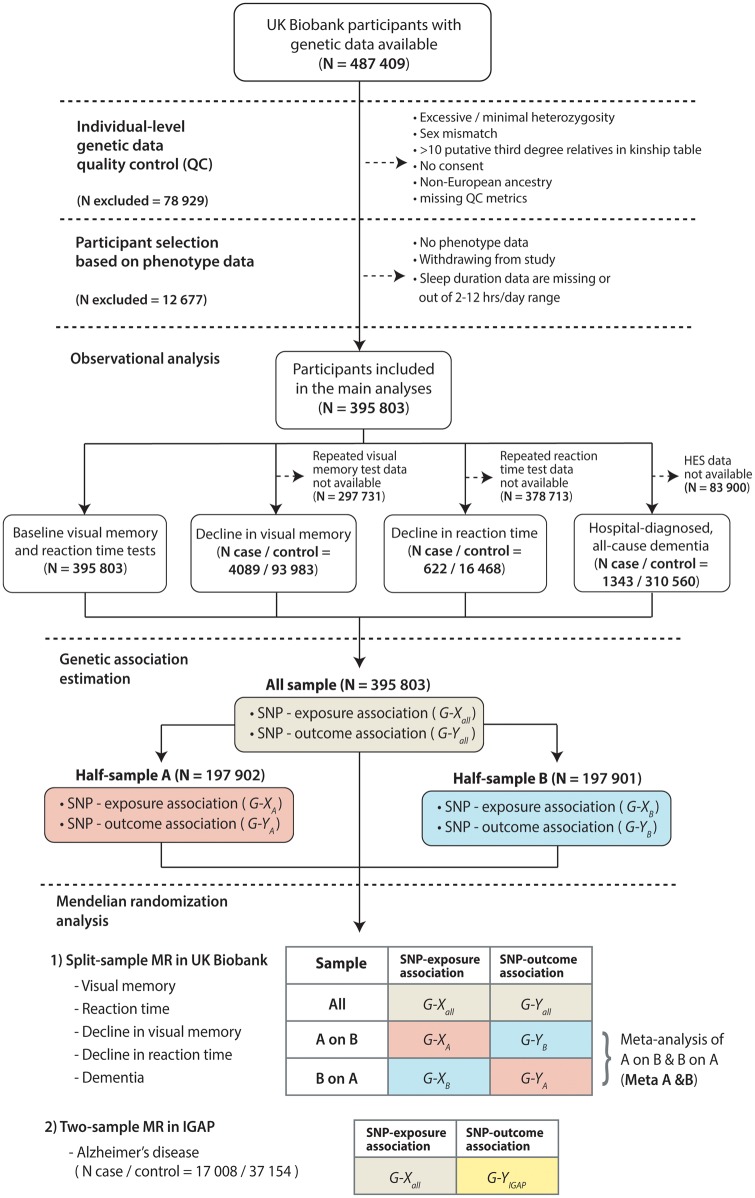
Study design. *N*, number of observations; HES, Hospital Episode Statistics; SNP, single-nucleotide polymorphism; MR, Mendelian randomization; G-X, genetic association of instrument (SNP) with exposure; G-Y, genetic association of instrument (SNP) with outcome; IGAP, International Genomics of Alzheimer’s Project.

UK Biobank is a large, population-based prospective cohort comprising linked health, hospital-record and genetic data of individuals aged 40–69 years recruited from across the UK between 2006 and 2010.^7^ Our main analyses included 395 803 UK Biobank participants. In the analyses for decline in visual memory (*N* case/non-case = 4089/93 983), decline in reaction time (622/16 468) and hospital-diagnosed all-cause dementia (*N* = 1343/310 560), we included only participants with repeated cognitive assessments and/or hospital-record data available. In the analyses for AD, we used summary statistics from a meta-analysis based upon genome-wide association studies (GWAS) (*N* case/control = 17 008/37 154) included in the IGAP stage I study (data were available at http://web.pasteur-lille.fr/en/recherche/u744/igap/igap_download.php).^8^ Details of participant selection are provided in Figure 1 and Supplementary Methods, available as Supplementary data at *IJE* online.

### Variable ascertainment

We used self-reported average sleep duration (hours/day) recorded at baseline as our exposure. We used results from baseline assessments of visual memory (number of errors made in pairs-matching test, natural log-transformed) and reaction time (milliseconds, natural log-transformed) as our continuous outcome variables. We used data from repeated assessments of visual memory and reaction time to derive binary cognitive decline variables (case or non-case) based on the standardized regression-based (SRB) method.^9^ We identified all-cause dementia cases based on previously validated primary and secondary ICD-10 diagnosis codes^10^ (Supplementary Table 1, available as Supplementary data at *IJE* online) from linked Hospital Episode Statistics (HES) data. We selected potential confounders based on previous literature,^2^^,^^3^ including sex, age, Townsend deprivation index, qualification, employment status, smoking status, alcohol-intake frequency, body mass index (BMI), systolic blood pressure, diastolic blood pressure, co-morbidities (Supplementary Table 2, available as Supplementary data at *IJE* online) and use of sleep-inducing medication (Supplementary Table 3, available as Supplementary data at *IJE* online).

### Genetic instrument selection

We took 78 near-independent SNPs for sleep duration with *P* for association <5 × 10^–^^8^ from a recent GWAS^11^ as our genetic instruments. Of these, one SNP (rs17761776) was excluded following SNP quality control (QC). Cumulatively, the remaining 77 SNPs in our genetic instruments explained 0.65% of the variability in sleep duration (*R*^2^ = 0.65%, *F*-statistic = 33.86). In this study, we used genotype dosage information to estimate allele count under an additive genetic model. More details on the instruments are provided in Supplementary Table 5, available as Supplementary data at *IJE* online. Information on SNP genotyping, imputation and QC are provided in Supplementary Methods, available as Supplementary data at *IJE* online.

### Statistical analyses

Figure 1 illustrates the design of this study.

### Observational analyses

We explored the observational association between sleep duration and each cognitive outcome using linear or logistic regression, with and without adjustment for potential confounders. Sleep duration was modelled as a discrete variable (ranging from 2 to 12 hours/day) and as a categorical variable (≤5, 6, 7, 8, 9, ≥10 hours/day). We performed analysis of variance (ANOVA) and chi-squared tests to compare means and proportions across sleep categories, and paired *t*-tests to assess within-individual differences for participants who completed both baseline and repeated cognitive assessments.

### Genetic-association analyses

Since the GWAS from which we identified our genetic instruments was conducted in UK Biobank,^11^ we used a split-sample strategy to mitigate the over-estimation of genetic effect sizes in one-sample setting (*winner’s curse bias*).^12^^,^^13^ We split the data randomly into two sets: A and B, with *N*_A_ = 197 902 and *N*_B_ = 197 901. We calculated individual SNP’s genetic association with exposure (*G-X*) and with outcome (*G-Y*) by running simple linear or logistic regressions in each set. For MR analyses, we used *G-X* from set A and *G-Y* from set B (*A on B*) and vice versa (*B on A*). Finally, we meta-analysed the MR estimates from the two (*Meta A & B*) and compared these to the estimate from the single-sample summary data (*All*). For AD, we used *G-X* estimated in our full UK Biobank sample and *G-Y* from IGAP stage I. Due to data unavailability, we used proxies for nine SNPs (linkage disequilibrium *R*^2^ > 0.9) and removed two SNPs without suitable proxy (rs34556183 and rs2139261). The remaining 75 SNPs had *R*^2^ = 0.64% and *F*-statistic = 33.91 in our UK Biobank sample.

### MR analyses

We applied the inverse-variance weighted (IVW) method as our main linear MR model. This method estimates the (linear) causal effect of the exposure on the outcome by averaging the genetic instruments’ ratio of instrument–outcome to instrument–exposure association estimates under a fixed-effect meta-analysis model.^14^ As sensitivity analyses, we ran MR-Egger regression^15^ and weighted median estimator (WME).^16^ The former produces an intercept term indicative for horizontal pleiotropy (where the genetic instruments are associated with the outcome through pathways other than the exposure)^15^ and the latter yields more robust estimates in the presence of some invalid genetic instruments.^16^

### Sensitivity analyses

We further explored the validity of our instruments by testing associations of potential confounders with the genetic score (constructed from summing genotype dosages across instruments), plotting genetic associations of each instrument with the exposure and the outcomes, and repeating our MR analyses with exclusion of potentially invalid instruments. In addition to the split-sample strategy, we also calculated the potential bias due to overlapping samples using a formula described elsewhere.^12^

### Non-linear MR

We investigated the non-linear associations of sleep duration with visual memory and reaction time using the piecewise linear MR method.^17^ Briefly, we stratified our sample into three strata based on the residual variation of the sleep duration after regressing on the genetic instruments. We then fitted a piecewise linear function in each stratum, which was constrained to be continuous, and took the gradient of each line segment as a localized average causal effect (LACE) in the stratum. Non-linearity was assessed using Cochran’s *Q* statistic for heterogeneity of the LACE estimates and test for quadratic exposure–outcome model.^17^ As sensitivity analysis, we re-ran the model with 10 strata using a de-discretized sleep-duration variable by adding small random variability through a series of Monte Carlo simulations.

We used R 3.4.3 and Stata 14 for data processing and statistical analyses. MR analyses and non-linear MR were performed using the *mrrobust* package in Stata^18^ and *nlmr* package in R,^17^ respectively. Further details of our methods are presented in Supplementary Methods, available as Supplementary data at *IJE* online.

## Results

### Baseline characteristics

Table 1 summarizes the baseline characteristics of study participants. The average sleep duration was 7.17 (1.07 SD) hours/day. We observed U-shaped/inverted U-shaped patterns across sleep-duration categories for most variables. Compared with participants who reported sleeping for 7 hours/day, both <7 and >7 hours/day sleep categories had lower scores in the baseline visual-memory and reaction-time tests, with those sleeping 10–12 hours/day scoring the worst [average number of incorrect matches = 4.6 (3.7 SD); average reaction time = 591 (134 SD) milliseconds].

**Table 1.** Characteristics of study participants

Variables	All participants	Sleep duration (hours / day)						*N*	*P*-value^a^
		≤5	6	7	8	9	≥10		
		*N* = 19 926	*N* = 73 813	*N* = 155 333	*N* = 116 573	*N* = 23 536	*N* = 6622		
		(5.0%)	(18.7%)	(39.3%)	(29.5%)	(6.0%)	(1.7%)		
**Baseline characteristics**									
Age (years), mean ± SD	56.9 ± 8	57.2 ± 7.7	56.6 ± 7.8	56.1 ± 8	57.3 ± 8.1	59 ± 7.8	58.7 ± 7.9	395 803	<0.001
Sex, %								395 803	<0.001
Female	54	56.5	52.3	52.5	56.3	56.5	55.2		
Male	46	43.5	47.7	47.5	43.7	43.5	44.8		
Townsend Deprivation Index, mean ± SD	−1.6 ± 2.9	−0.7 ± 3.3	−1.4 ± 3	−1.7 ± 2.8	−1.7 ± 2.8	−1.6 ± 2.9	−0.7 ± 3.3	395 803	<0.001
College/university/professional qualification, %	36.4	24.6	34	40.5	36.5	30.5	23.6	395 803	<0.001
**Employment status, %**								395 803	<0.001
Employed	57.1	50.8	62.2	64.5	51.3	35.5	24		
Retired	35.1	34.2	30.1	29.6	41	53.6	51.7		
Others	7.8	15	7.7	5.9	7.7	10.8	24.3		
Smoking status, %								395 803	<0.001
Never	54.7	49.9	52.7	56.4	55.5	52	46.6		
Previous	35.3	34.9	35.8	34.5	35.5	37.7	38.4		
Current	10	15.2	11.5	9.2	9	10.3	14.9		
Alcohol consumption, %								395 803	<0.001
Rarely	27.9	38.5	29.4	25.3	27.3	31.2	41.5		
1–2 a week	26.4	25.4	26.6	26.8	26.5	25.1	23		
3–4 a week	24.3	18.6	23.2	26.2	24.4	21.6	15.9		
Almost daily	21.3	17.5	20.8	21.7	21.8	22.1	19.6		
BMI (kg/m^2^), mean ± SD	27.4 ± 4.7	28.5 ± 5.4	27.8 ± 4.9	27.1 ± 4.5	27.2 ± 4.6	27.8 ± 4.9	29.1 ± 5.7	395 803	<0.001
SBP (mmHg), mean ± SD	138 ± 19	139 ± 19	138 ± 18	138 ± 18	139 ± 19	140 ± 19	139 ± 19	373 248	<0.001
DBP (mmHg), mean ± SD	82 ± 10	83 ± 10	82 ± 10	82 ± 10	82 ± 10	83 ± 10	83 ± 10	373 251	<0.001
Co-morbidities present, %	38.7	49.3	39.8	35.2	37.8	47.3	63.6	395 803	<0.001
Use of sleep-inducing medication, %	1.1	3.4	1.2	0.7	0.9	1.5	4.2	395 803	<0.001
**Cognitive outcomes**									
Baseline cognitive outcomes (all participants)									
VM, mean ± SD	4.1 ± 3.2	4.2 ± 3.3	4 ± 3.2	4 ± 3.1	4.1 ± 3.3	4.3 ± 3.4	4.6 ± 3.7	395 803	<0.001
RT, mean ± SD	555 ± 113	566 ± 122	554 ± 113	549 ± 109	558 ± 113	569 ± 116	591 ± 134	395 803	<0.001
**Repeated VM assessment**								98 072	
VM (baseline), mean ± SD	3.7 ± 2.9	3.9 ± 3	3.8 ± 2.9	3.7 ± 2.9	3.8 ± 2.9	3.9 ± 3	3.9 ± 2.9		<0.001
VM (repeated), mean ± SD	4.2 ± 3.1	4.3 ± 3.3	4.2 ± 3.1	4.1 ± 3	4.2 ± 3.1	4.3 ± 3.1	4.3 ± 3.2		<0.001
Decline in VM case, %	4.2	4.8	4.3	4	4.2	4.4	4.3		0.24
**Repeated RT assessment**								17 090	
RT (baseline), mean ± SD	548 ± 103	552 ± 114	546 ± 99	544 ± 101	552 ± 105	555 ± 97	582 ± 121		<0.001
RT (repeated), mean ± SD	556 ± 109	561 ± 110	554 ± 109	552 ± 108	558 ± 112	569 ± 103	580 ± 114		<0.001
Decline in RT case, %	3.6	3.7	3.7	3.6	3.5	5	1.9		0.16
Dementia, %	0.43	0.67	0.39	0.31	0.43	0.71	1.5	311 903	<0.001

a *P*-value from ANOVA/chi-squared tests comparing mean/proportion across sleep categories.

VM, visual memory (score reflects number of errors made in pairs-matching test); RT, reaction time (score reflects time to react in millisecond); Decline in VM / RT, decline in visual memory / reaction time derived from standardized regression-based method; BMI, body mass index; SBP, systolic blood pressure; DBP, diastolic blood pressure; *N*, total number of observations (for binary outcomes; *N* includes both cases and non-cases).

We identified 4089 (4.2%, from a total of *N*_total_ = 98 072) participants with decline in visual memory, 622 (3.6%, *N*_total_ = 17 090) with decline in reaction time and 1343 (0.43%, *N*_total_ = 311 903) diagnosed with dementia. On average, performance in repeated assessments was poorer than baseline for both visual-memory [baseline mean = 3.7 (2.9 SD); repeated mean = 4.2 (3.1 SD); *P *<* *0.001] and reaction-time tests [baseline mean = 548 (103 SD) milliseconds; repeated mean = 556 (109 SD) milliseconds; *P *<* *0.001]. Participants diagnosed with dementia performed worse than those without the disease in baseline cognitive tests [average number of incorrect matches = 5.1 (4.2 SD), *P *<* *0.001; average reaction time = 635 (157 SD) milliseconds, *P *<* *0.001].

### Observational analyses

Table 2 outlines the results from observational analyses with categorical sleep duration. For the log-transformed cognitive assessment results, we report exponentiated betas [Exp(β)] to ease interpretation. The Exp(β) represent a multiplicative effect size, e.g. Exp(β) = 1.03, in reaction-time test, which represents an estimated *Exp(*β*)* *–* *1 *=* *0.03 = 3% slower reaction time. On average, individuals who reported sleep for less or more than 7 hours/day had more incorrect matches in baseline visual-memory test, slower baseline reaction time and increased risk of dementia, but had little to no difference in the risk of cognitive decline. These associations were attenuated upon adjustment for potential confounders.

**Table 2.** Observational associations with categorical sleep duration

Outcomes	*N* observation or *N* case/non-case	Sleep duration in categories (hours/day)					
		≤5	6	7	8	9	≥10
**Unadjusted model**							
Baseline cognitive assessment, exponentiated beta (95% CI)							
Visual memory	395 803	1.04 (1.03, 1.05)*	1.01 (1.00, 1.02)**	Ref	1.03 (1.02, 1.03)*	1.05 (1.05, 1.06)*	1.11 (1.09, 1.12)*
Reaction time	395 803	1.03 (1.02, 1.03)*	1.01 (1.01, 1.01)*	Ref	1.02 (1.12, 1.02)*	1.03 (1.03, 1.04)*	1.07 (1.07, 1.08)*
Binary cognitive outcomes, OR (95% CI)							
Decline in visual memory	4089/93 983	1.19 (1.01, 1.41)**	1.07 (0.98, 1.17)	Ref	1.04 (0.96, 1.12)	1.09 (0.95, 1.26)	1.06 (0.78, 1.44)
Decline in reaction time	622/16 468	1.03 (0.67, 1.59)	1.04 (0.83, 1.31)	Ref	0.97 (0.80, 1.18)	1.43 (1.05, 1.94)**	0.53 (0.20, 1.44)
Dementia	1343/310 560	2.14 (1.73, 2.64)*	1.26 (1.07, 1.49)**	Ref	1.39 (1.20, 1.60)*	2.28 (1.88, 2.78)*	4.85 (3.84, 6.12)*
**Adjusted model** ^a^							
Baseline cognitive assessment, exponentiated beta (95% CI)							
Visual memory	395 803	1.02 (1.01, 1.03)*	1.00 (1.00, 1.01)	Ref	1.01 (1.00, 1.01)*	1.02 (1.01, 1.02)*	1.06 (1.05, 1.08)*
Reaction time	395 803	1.00 (1.00, 1.01)*	1.00 (1.00, 1.00)	Ref	1.00 (1.00, 1.00)*	1.00 (1.00, 1.01)*	1.03 (1.02 1.03)*
Binary cognitive outcomes, OR (95% CI)							
Decline in visual memory	4089/93 983	1.19 (1.01, 1.41)**	1.08 (0.99, 1.18)	Ref	1.01 (0.94, 1.09)	1.04 (0.90, 1.20)	1.03 (0.76, 1.40)
Decline in reaction time	622/16 468	0.93 (0.60, 1.45)	1.02 (0.81, 1.28)	Ref	0.92 (0.76, 1.12)	1.26 (0.93, 1.72)	0.43 (0.16, 1.18)
Dementia	1343/310 560	1.54 (1.24, 1.91)*	1.15 (0.97, 1.36)	Ref	1.12 (0.97, 1.29)	1.39 (1.14, 1.69)**	2.28 (1.79, 2.90)*

* *P *<* *0.001; **P <* *0.05.

a Adjusted for age, sex, socio-economic status, qualification, employment, smoking status, alcohol-intake frequency, body mass index, hypertension, co-morbidities and use of sleep-inducing medication.

OR, odds ratio; 95% CI, 95% confidence interval; numbers represent effect size per additional hour/day in sleep duration; visual memory was measured as natural log of (numbers of errors in pairs-matching test + 1); reaction time was measured as natural log of milliseconds reaction time; exponentiated beta represents a multiplicative effect size (as the outcomes were log-transformed), e.g. an exponentiated beta of 1.03 in reaction time represents an estimated 3% increase in reaction-time test (3% slower).

### MR analyses

**Figure 2.**
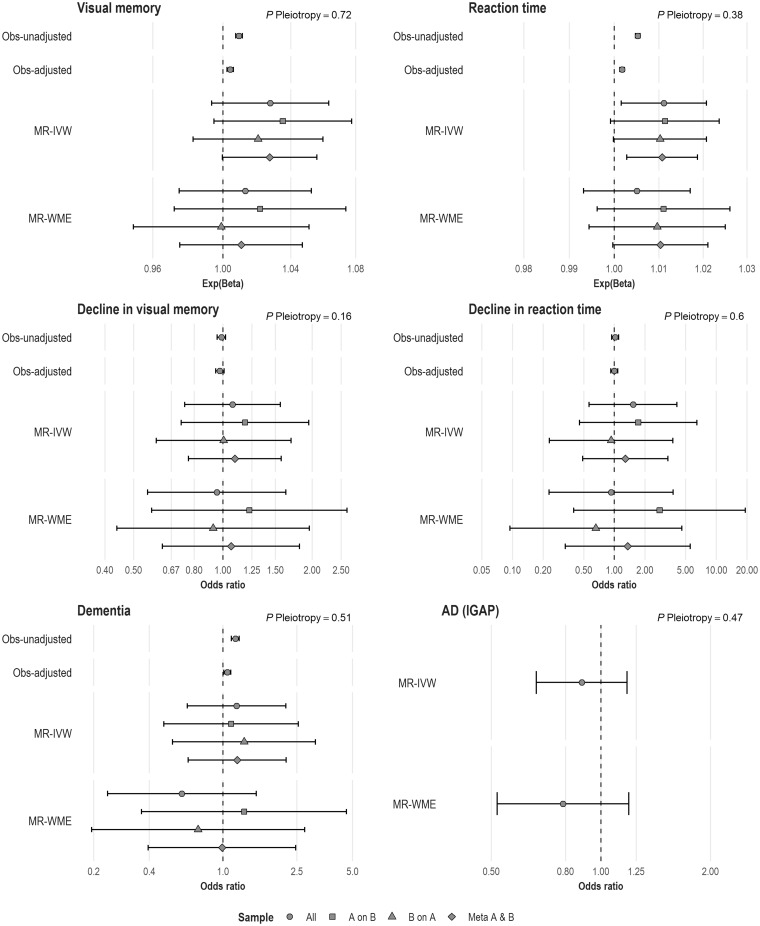
Results from observational and Mendelian randomization analyses. Numbers represent effect size per additional hour/day of sleep duration; Exp(Beta), exponentiated beta (represents multiplicative effect size, e.g. an exponentiated beta of 1.03 in reaction time represents an estimated 3% increased/slower reaction time); *P* Pleiotropy, *P*-value for overall horizontal pleiotropic effect as indicated by the intercept from MR-Egger regression; Obs-unadjusted, unadjusted observational analysis; Obs-adjusted, observational analysis adjusted for age, sex, socio-economic status, qualification, employment, smoking status, alcohol-intake frequency, body mass index, hypertension, co-morbidities and use of sleep-inducing medication; MR-IVW, Mendelian randomization, inverse-variance-weighted; MR-WME, Mendelian randomization, weighted median estimator.

Comparisons between the observational and the MR analyses for linear sleep duration are summarized in Figure 2. Full estimates are provided in Supplementary Table 6, available as Supplementary data at *IJE* online.

Linear MR analyses revealed that each additional hour/day in sleep duration was associated with an estimated 1% slower reaction time {exponentiated beta from IVW method in meta-analysis sample – Exp(β)_IVW-meta_ = 1.01 [95% confidence interval (CI) = 1.00 – 1.02]; *P* = 0.008}. The evidence for an association with visual memory was directionally consistent [Exp(β)_IVW-meta_ = 1.03 (95% CI = 1.00–1.06); *P *=* *0.05]. These estimates were similar to observational analysis results.

In both observational and linear MR analyses, we found no evidence of an association with the risk of prospective cognitive decline in visual memory [odds ratio per additional hour/day in sleep duration for the IVW method in our meta-analysis sample– OR_IVW-meta_ = 1.10 (95% CI = 0.76–1.57); *P *=* *0.62] or reaction time [OR_IVW-meta_ = 1.28 (95% CI = 0.49–6.49)].

Observational data suggested some evidence of an association with dementia [OR in adjusted model = 1.05 (95% CI = 1.01–1.10); *P *=* *0.02]. Findings from linear MR-IVW analysis were directionally consistent, but imprecise [OR_IVW-meta_ = 1.19 (95% CI = 0.65–2.19); *P *=* *0.57]. Similarly, we found no evidence of an association between sleep duration and the risk of AD in IGAP [OR_IVW_ = 0.89 (95% CI = 0.67–1.18); *P *=* *0.41].

### Sensitivity analyses

In our linear MR analyses, both IVW and WME methods produced broadly consistent results, with MR-Egger intercept *P-*values ranging from 0.16 to 0.72, suggesting no horizontal pleiotropy effect (Supplementary Figure 1, available as Supplementary data at *IJE* online).

We found several associations of our genetic score with other variables, including BMI, co-morbidities and some lifestyle factors (*P *<* *0.003, accounting for multiple testing), which we hypothesized might be partly driven by rs9940646, a marker in the FTO gene (widely recognized to be associated with BMI and obesity^19^). Exclusion of this variant from our genetic score did not completely diminish these associations (Supplementary Table 7, available as Supplementary data at *IJE* online), but produced consistent MR estimates (Supplementary Table 6, available as Supplementary data at *IJE* online).

We estimated that the biases due to sample overlap were small (absolute value of bias <0.005 for all outcomes) with type-1 error rate = 0.05 (Supplementary Table 8, available as Supplementary data at *IJE* online).

### Non-linear MR analyses

**Figure 3.**
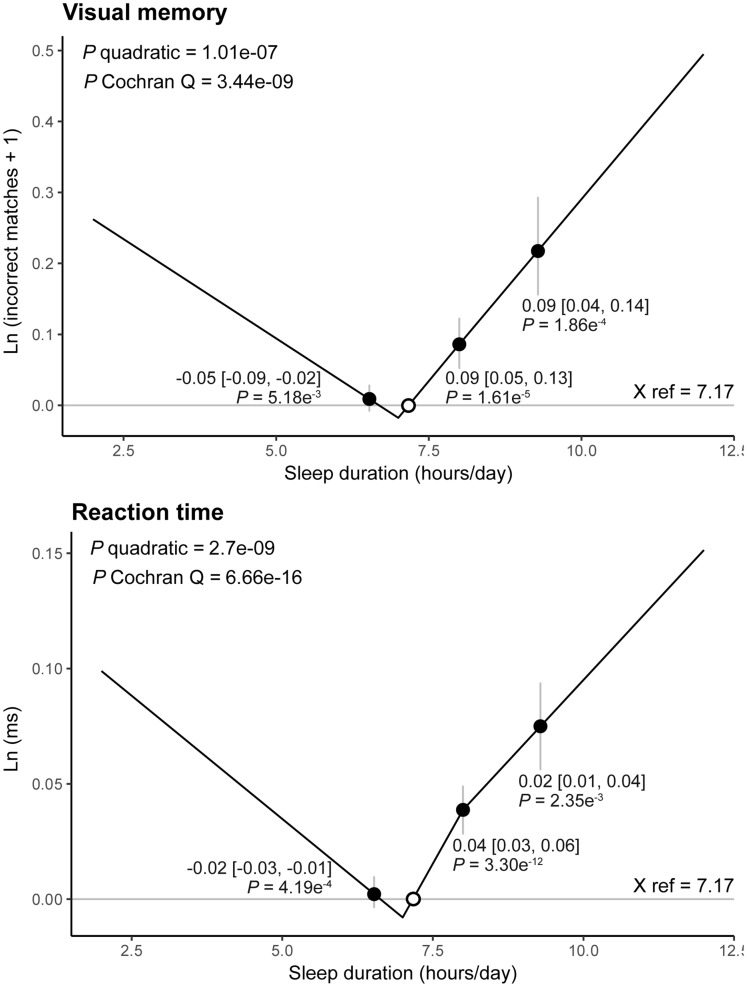
Non-linear Mendelian randomization results with piecewise linear method using three strata of sleep duration conditioned on the genetic instruments. Annotated numbers [black dots (grey vertical lines)] represent localized average causal effect (95% confidence interval) in each stratum; white dots, mean sleep duration used as reference point (X ref); *P* quadratic/Cochran Q, *P*-value for non-linearity from quadratic/Cochran Q test; *Ln* (incorrect matches + 1), natural log of [number of incorrect matches (errors made) in visual-memory test + 1]; *Ln* (ms), natural log milliseconds of reaction time.

The piecewise linear MR with three strata (Figure 3) suggested evidence of non-linear associations of sleep duration with both visual memory (quadratic test *P *=* *1.01e^–^^7^, Cochran Q test *P *=* *3.44e^–^^9^) and reaction time (quadratic test *P *=* *2.7e^–^^9^, Cochran Q test *P *=* *6.66e^–^^16^). In both outcomes, the absolute value for LACE estimates in the long-sleep-duration strata were higher (steeper slope in Figure 3) than in the short-sleep-duration strata, suggesting a J-shaped association. This was supported by findings from experimental simulations with 10 strata (Supplementary Figure 2A and B, available as Supplementary data at *IJE* online).

## Discussion

Using MR, we found that a linear increase in sleep duration was associated with a small reduced performance in reaction-time and visual-memory tests. This small linear-effect size may indicate that the true association is non-linear, as demonstrated in our non-linear MR model. Whilst the underlying pathways accounting for these associations remain to be elucidated, our findings suggest that sleep duration may represent a potential modifiable risk factor for cognition in mid-life, for which effective pharmacological interventions are currently lacking.

Both short and long sleep duration have been associated with worse cognitive outcomes in previous observational reviews.^2^^,^^3^ These associations were confirmed in our observational analyses and supported by the findings from our non-linear MR analyses. Results from linear and non-linear MR suggest that the causal effect in the long-sleeper group was larger than the short-sleeper group (J-shaped association), consistently with that of a recent meta-analysis^20^ and a cross-sectional study using objectively measured sleep duration.^21^

Sleep duration is inextricably linked with sleep quality^22^ and poor sleep quality could disrupt the circadian rhythm, which regulates gene expression in the frontal, thalamic and hypothalamic regions and the brainstem locus coeruleus.^23^ This might impair neurogenesis^24^ and hippocampal function^25^—region that shows early alteration in several neurodegenerative process leading to cognitive dysfunction. Disordered sleep may have different effects on brain functions linked with specific cognitive domains, e.g. synchronization function of the prefrontal cortex and neuromodulatory system in visual memory^26^ or the prefrontal cortex and cerebellar functions in reaction time.^27^

Similarly, short and long sleep duration^28–30^ and poor sleep quality^31^ have also been linked with an increased risk of dementia. Although a similar J-shaped association was observed in our observational analysis, we were limited to performing only the linear MR analysis, as the non-linear MR method requires a large number of cases and individual-level data. In our linear MR analysis, we found no clear evidence that an increased sleep duration was associated with a higher risk of all-cause dementia in UK Biobank or with AD in IGAP. This is unsurprising, as the true association might be non-linear and we were limited with only 1343 dementia cases in UK Biobank. Also, IGAP does not capture non-AD dementia types and comprises an older and more heterogeneous population.^8^

The main strength of our study lies in the MR analysis, which minimizes residual confounding and reverse causation.^2^ The use of genetic instruments allowed us to estimate a life-long effect of sleep duration on the outcomes and the inclusion of multiple genetic instruments enabled increased power for MR analysis, mitigating weak instrument bias.^32^ Pleiotropic effects were carefully explored and minimized through MR-Egger analysis, WME and investigation of the effect of individual SNPs. In order to mitigate the potential inflated type-I error rate due to overlapping samples,^12^ we used a split-sample strategy and found that meta-analysed estimates for both visual memory and reaction time were similar to the single-sample estimate. Moreover, we attempted to quantify the bias^12^ assuming 100% sample overlap and found it to be small.

Another important strength is that we are one of the first studies to implement non-linear MR analyses and, importantly, these results were consistent with findings from both observational and linear MR analyses, helping to provide better insight into the nature of the association. However, these findings should be interpreted carefully, as sleep duration was only available as a discrete variable in our dataset, which resulted in sub-optimal stratification in our non-linear MR model. Whilst we attempted to improve this by de-discretizing our exposure and found consistent J-shaped associations through simulations, ideally our analysis should be replicated with a more precise continuous measurement of sleep duration (e.g. with actigraphy).

Other limitations include potential reliability issues with the partly novel cognitive assessments and self-reported sleep duration in UK Biobank. However, the cognitive assessments have been validated^33^ and we also found that lower scores were more frequent in people with dementia. As for sleep duration, self-reported assessment might be more relevant especially in primary health-care settings for practical reasons.^34^ The MR estimates for prospective cognitive decline were imprecise due to the limited number of cases and practice effects^33^ may have influenced the reliability of the repeated assessments. Whilst the SRB method can mitigate this issue,^9^ another method to define cognitive decline could be applied, e.g. by calculating a smallest real-difference cut-off point.^33^ In addition, the time between assessments in our sample [mean = 5.8 (0.8 SD) years for visual memory; 4.3 (0.9 SD) years for reaction time] might be not long enough for cognitive decline to manifest. Additionally, there may be selection bias in UK Biobank due to low response rates.^33^

Each of the associations of our genetic score with potential confounders warrants further investigation, but is beyond the scope of this paper. As many of these traits have been widely recognized to be polygenic in nature, they may share some common genetic architecture with sleep duration. Alternatively, these associations may represent downstream effects from sleep duration (i.e. vertical pleiotropy) that do not violate MR assumptions.

In summary, this study provides novel evidence that increased sleep duration may be causally related to poorer reaction time and poorer visual memory, albeit with relatively small linear-effect sizes. The true associations might be J-shaped for both outcomes, but this remains to be confirmed with a more precise sleep-duration measurement. Results for risks of dementia and AD are still too imprecise to draw any definitive conclusions. Our findings suggest that, in clinical care, attention should be paid to sleep-duration patterns and improved sleep habits could represent a potential therapeutic target for cognition. This seems important, as, currently, no single-measure treatment has been shown to decelerate cognitive decline or the risk of dementia. Lastly, we would recommend that most healthy adults should aim to follow the recommendation of 7–9 hours of sleep per day^35^ and also pay attention to long-term changes in sleep patterns.^36^

## Funding

This work was supported by various funders. A.H. was supported by the Indonesian Endowment Fund For Education (awardee ID 20160412045979); M.K. was supported by the British Heart Foundation (FS/18/5/33319). S.D. was supported by the National Institute for Health Research (RP-PG-0407–10314), Wellcome Trust (086091/Z/08/Z) and the Farr Institute of Health Informatics Research, funded by the Medical Research Council (MR/K006584/1), in partnership with Arthritis Research UK, the British Heart Foundation, Cancer Research UK, the Economic and Social Research Council, the Engineering and Physical Sciences Research Council, the National Institute of Health Research, the National Institute for Social Care and Health Research (Welsh Assembly Government), the Chief Scientist Office (Scottish Government Health Directorates) and the Wellcome Trust. V.G. was supported by a multidisciplinary grant from the Economic and Social Research Council and Medical Research Council (grant number ES/J500185/1). C.D. was supported by a UCL Springboard Population Science fellowship (grant number 105604/Z/14/Z) funded by the Wellcome Trust. This study used the UK Biobank resources that has been funded by the Wellcome Trust Medical Charity, Medical Research Council, Department of Health of Scottish Government, the Northwest Regional Development Agency, the Welsh Assembly Government and the British Heart Foundation.

## Supplementary Material

dyz071_Supplementary_Materials
