# Supplementary material for: The relationship between sleep duration, cognition and dementia: a Mendelian randomization study
Source: Int J Epidemiol. 2019 May 7;48(3):849–60. doi: 10.1093/ije/dyz071 (PMC6659373; doi:10.1093/ije/dyz071)
Supplement: dyz071_Supplementary_Materials [file dyz071_supplementary_materials.zip › dyz071-suppl_data/Supplementary Methods.docx]

**SUPPLEMENTARY METHODS**

The relationship between sleep duration, cognition, and dementia: A Mendelian randomization study

**AUTHORS**

Albert Henry^1,2*^, Michail Katsoulis^1^, Stefano Masi^2,3^, Ghazaleh Fatemifar^1^, Spiros Denaxas^1^, Dionisio Acosta^1^, Victoria Garfield^2†^, Caroline E. Dale^1,2†^

* First author, corresponding author

^†^ Joint last authors

**AFFILIATIONS**

1. Institute of Health Informatics, University College London, London, United Kingdom
2. Institute of Cardiovascular Science, University College London, London, United Kingdom
3. Department of Clinical and Experimental Medicine, University of Pisa, Pisa, Italy

**CORRESPONDING AUTHOR**

Albert Henry

Institute of Health Informatics

University College London

222 Euston Road, NW1 2DA

London, United Kingdom

Phone: +44 (0) 2031086723

E-mail: [albert.henry.16@ucl.ac.uk](mailto:albert.henry.16@ucl.ac.uk)

## **Participant selection**

## At the time of our study, UK Biobank had genetic data available for 487 409 participants. We applied individual-level quality control (QC) to exclude participants with excessive / minimal heterozygosity, sex mismatch, excessive genetic relatedness (more than 10 putative third-degree relatives in the kinship table), no consent, non-European ancestry, and missing QC metrics, leaving 408 480 participants for analysis. We further excluded participants with no phenotype data (N = 41), withdrawing from study (N = 5), and sleep duration data are missing or out of 2­–12 hours/day range (N = 12 631), leaving 395 803 participants for subsequent analyses. The analyses for decline in visual memory (N case / non-case =4089 / 93 983), decline in reaction time (N case / non-case =622 / 16 468), and hospital-diagnosed, all-cause dementia (N case / non-case =1343 / 310 560) included only participants with repeated cognitive assessments and/or hospital record data available (**Figure 1**).

**Variable ascertainment**

**Sleep duration**

Sleep duration was recorded at baseline using self-reported questionnaire as the average hours of sleep (including naps) in 24 hours, or the average in the last 4 weeks if it varies.

**Cognitive outcomes**

*Baseline cognitive assessment*

The UK Biobank administered five baseline cognitive tests through computerised touch-screen interface^1^. In this manuscript, we focus on visual memory and reaction time tests as these two measures have the biggest sample size to enable MR analysis. In the visual memory test, participants were asked to identify matches from six pairs of cards after memorising their positions. The number of incorrect matches (errors made) was recorded and a higher number indicates poorer visual memory. Reaction time was measured as mean time (in milliseconds) taken to correctly identify matches from 12 rounds of the card game ‘Snap’, where a longer time reflects a slower reaction. Due to a positively skewed distribution, both test results were transformed using natural logarithmic functions *ln(x)* for reaction time test and *ln(x+1)* for visual memory test (with the constant added to account for zero inflation).

*Prospective cognitive decline*

We used data from repeated assessments in 2012­–2013 for reaction time (N =17 090) and from online follow-up in 2014–2015 for visual memory (N =98 072) to derive binary cognitive decline variables (case or non-case) using *Standardised Regression Based* (SRB) method^2^. We used information on baseline assessment, sex, age, qualifications, and time between assessments as predictors for repeated assessment in a multivariate regression model. Cognitive decline was assigned to individuals whose standardised residual values were larger than 1.96 (representing a type-I error rate of 0.05).

*Dementia*

We identified all-cause dementia cases based on previously validated primary and secondary ICD-10 diagnosis codes^3^ (**STable 1**) from linked Hospital Episode Statistics (HES) data, which contain structured administrative records of all admissions, outpatient appointments, and emergency attendances at National Health Services hospitals in England.

**Confounders**

Potential confounders were identified based on previous literature^4,5^. Information on participants’ sleep duration, age, sex, qualifications, employment status, smoking status, and alcohol intake frequency were collected using a self-reported questionnaire at baseline. Townsend deprivation index was assigned based on postcode as a continuous measure, where a higher index indicates more deprivation. Qualifications were dichotomised into college / university graduate / professional or not, based on participants’ highest qualification. Current employment status was divided into three categories: employed (including paid and self-employment), retired, or others. For alcohol consumption, participants who never consume alcohol, only drink on special occasions, and drink 1­–3 times a month were grouped into the ‘rarely’ category. Body mass index was calculated as weight (kg) / height (m)^2^ from physical examination data. Systolic diastolic blood pressure were calculated from the average of the two baseline recordings. Comorbidities (including cardiovascular, respiratory, metabolic, neurology, and psychiatric illnesses) were identified as a binary variable from baseline self-reported illness data using UK Biobank code list (**STable 3**). Use of sleep-inducing medication was defined as regular use of drugs listed under hypnotics and anxiolytics categories in the British National Formulary treatment summary^6^, derived from baseline self-reported medication data. Since the raw data were unstructured, we considered only drugs with ≥50 users to derive this variable (**STable 4**).

**Genotyping, Imputation, and quality control**

Genotyping in UK Biobank was performed using two customised genome-wide arrays, with a genome-wide imputation performed using combined reference panels from UK10K, 1000 Genomes phase 3, and Haplotype Reference Consortium (HRC), resulting in 93 095 623 autosomal variants^7^. We further applied quality control to exclude variants with the following criteria: Fisher information <0.3, missing call rate ≥ 5%, or MAF outside of 0.01–1 range. The IGAP stage I meta-analysis included 7 055 881 SNPs, from genotyping and imputation using European population reference in 1000 Genomes 2010 interim release dataset^8^.

**Mendelian randomization (MR) analysis methods**

MR analysis uses genetic variants as proxies for exposure of interest to estimate the causal association between the exposure and outcome of interest in an instrumental variable (IV) framework. A conventional MR makes assumptions that genetic instruments for the exposure must be:

- Reliably associated with the exposure
- Associated with the outcome only through exposure, and
- Independent of unobserved confounders that influence the exposure and outcome after conditioning on observed confounders^9^

Variation of MR study design and method has been discussed in details elsewhere^9,10^. In this study, we focus on multi-instrument, two-sample MR (where summary statistics for genetic associations with the exposure and the outcome, typically estimated in two independent samples, were used to calculate the MR estimate). Specifically, we used the following methods:

1. *Inverse-variance weighted*

For uncorrelated genetic variant $j$, the causal estimate $\hat{\beta}_{IVW}$ can be calculated by averaging the ratio between variant-outcome association estimate $\hat{\beta}_{Y_{j}}$ and variant-exposure association estimate $\hat{\beta}_{X_{j}}$ using the inverse-variance weighted formula for fixed-effect meta-analysis model, which yields:

$$\hat{\beta}_{IVW}= \frac{\sum_{j} \hat{\beta}_{Y_{j}}\hat{\beta}_{X_{j}}\sigma_{Y_{j}}^{-2}}{\sum_{j} \hat{\beta}_{X_{j}}^{2}\sigma_{Y_{j}}^{-2}}$$

where $\hat{\beta}_{Y_{j}}$ (or $\hat{\beta}_{X_{j}}$) and $\sigma_{Y_{j}}$ are the coefficient and standard error from regression of cognitive outcomes $Y$ (or sleep duration $X$) on variant $j$^11^.

1. *MR-Egger regression*

The MR-Egger regression performs a weighted linear regression of $\hat{\beta}_{Y_{j}}$ on the $\hat{\beta}_{X_{j}}$, using the $\sigma_{Y_{j}}^{-2}$ as weights and with unconstrained intercept^11^. The causal estimate $\hat{\beta}_{E}$ is obtained from the model:

$$\hat{\beta}_{Y_{j}}= \hat{\alpha}_{E}+\hat{\beta}_{E} \hat{\beta}_{X_{j}}$$

The intercept term $\hat{\alpha}_{E}$ denotes the estimated horizontal pleiotropic effect across genetic variants, and thus, the associated *P*-value is indicative of overall horizontal pleiotropy. As the MR-Egger estimates typically have low power, we only used this method to detect horizontal pleiotropy^12^.

Both the IVW and MR-Egger models make several additional assumptions, including NO Measurement Error (NOME), InSIDE (Instrument Strength Independent of Direct Effect), and VIS (Variation in Instrument Strength), as detailed elsewehere^13^.

1. *Weighted median estimator*

In the weighted median estimator, first the causal estimate $\hat{\beta}$ of each variant $j$ is calculated with the ratio method as $\hat{\beta}_{j}={\hat{\beta}_{Y_{j}}}/{\hat{\beta}_{X_{j}}}$. Then, $\hat{\beta}_{j}$ are sorted (so that $\hat{\beta}_{1}< \hat{\beta}_{2}<\ldots< \hat{\beta}_{J}$) and standardised weight $w_{j}$ is assigned to the $j$th-ordered ratio estimate. The weights are calculated using the inverse variance of the ratio estimates as $\acute{w}_{j}=\hat{\beta}_{X_{j}}\sigma_{Y_{j}}^{-2}$. The standardised weights are $w_{j}={\acute{w}_{j}}/{\sum_{j} \acute{w}_{j}}$ and their sum is $s_{j}= 1$.

If $k$ denotes the largest integer such that the sum of weights up to and including the $k$th estimate ($s_{k}= \sum_{j\leq k} w_{j}$ ) is <0.5, the causal estimate from weighted median method $\hat{\beta}_{WME}$ can be calculated by interpolation between the $k$th and $(k+1)$th ratio estimates as follows:

$$\hat{\beta}_{WME}= \hat{\beta}_{k}+\left( \hat{\beta}_{k+1}- \hat{\beta}_{k} \right) \times\frac{0.5- s_{k}}{s_{k+1}- s_{k}}$$

This approach should provide a consistent estimate given that at least 50% of the weights are derived from valid variants and more robust to violation of the untestable InSIDE assumption^14,15^.

**Non-linear MR**

We applied the non-linear MR with piece-wise linear method^16^ to estimate non-linearity of the associations of sleep duration with visual memory and reaction time. With this approach, we calculate the exposure‐outcome relationship as piece-wise linear function with each stratum contributing a line piece whose gradient is the localized average causal effect estimate (LACE) for that stratum. This function is constrained to be continuous, so that each line piece starts where the previous one ends. However, this approach had an important limitation in our dataset since the sleep duration exposure was a discrete variable (ranging from 2 to 12 hours/day) rather than truly continuous.  The limited number of unique values in sleep duration means that there are limited variability in some of the strata, and consequently the model fails when the exposure within a stratum have constant values.

To overcome this limitation, first we ran the model with only 3 strata (maximum numbers of strata for which model converged) to identify the overall shape of the association (**Figure 3**). Next, we attempted to transform the discrete sleep duration variable into continuous variable by generating a random number drawn from the normal distribution with the initial value as the mean and an arbitrary standard deviation of 0.1 through Monte Carlo simulation. This approach enabled us to run the model with more strata and to inspect the shape of the association more clearly. We ran ten simulations with ten strata for each outcome and observed that the results are consistent (**SFigures 2A-B**) – the inference remains the same as when using 3 strata only with original discrete exposure. Lastly, we tested the non-linear hypothesis using Cochran’s *Q* statistic for heterogeneity of the LACE estimates, and metaregression of the LACE estimates against the mean value of the exposure in each strata (equivalent to fitting a quadratic exposure‐outcome model)^16^.

# **Supplementary References**

1. Lyall DM, Cullen B, Allerhand M, et al. Cognitive test scores in UK biobank: Data reduction in 480,416 participants and longitudinal stability in 20,346 participants. *PLoS One*. 2016;**11**(4):1–10.

2. Frerichs RJ, Tuokko HA. A comparison of methods for measuring cognitive change in older adults. *Arch Clin Neuropsychol*. 2005;**20**(3):321–333.

3. Pujades-Rodriguez M, Assi V, Gonzalez-Izquierd A, et al. The diagnosis, burden and prognosis of dementia: a record-linkage cohort study in England and Wales. *PLOS ONE (Under Rev*.

4. Devore EE, Grodstein F, Schernhammer ES. Sleep duration in relation to cognitive function among older adults: A systematic review of observational studies. *Neuroepidemiology*. 2016;**46**(1):57–78.

5. Lo JC, Groeger JA, Cheng GH, Dijk D-J, Chee MWL. Self-reported sleep duration and cognitive performance in older adults: a systematic review and meta-analysis. *Sleep Med* [Internet]. Elsevier B.V.; 2016;**17**:87–98. Available from: http://linkinghub.elsevier.com/retrieve/pii/S1389945715019796

6. BNF. BNF Treatment Summary - Hypnotics and Anxiolytics [Internet]. 2017. Available from: https://bnf.nice.org.uk/treatment-summary/hypnotics-and-anxiolytics.html

7. Bycroft C, Freeman C, Petkova D, et al. The UK Biobank resource with deep phenotyping and genomic data. *Nature* [Internet]. Nature Publishing Group; 2018 Oct 10 [cited 2018 Dec 5];**562**(7726):203–209. Available from: http://www.nature.com/articles/s41586-018-0579-z

8. Lambert J-C, Ibrahim-Verbaas CA, Harold D, et al. Meta-analysis of 74,046 individuals identifies 11 new susceptibility loci for Alzheimer’s disease. *Nat Genet* [Internet]. Nature Publishing Group, a division of Macmillan Publishers Limited. All Rights Reserved.; 2013 Dec;**45**(12):1452–1458. Available from: http://dx.doi.org/10.1038/ng.2802

9. Davey Smith G, Hemani G. Mendelian randomization: genetic anchors for causal inference in epidemiological studies. *Hum Mol Genet* [Internet]. 2014;**23**(R1):R89-98. Available from: http://www.ncbi.nlm.nih.gov/pubmed/25064373%5Cnhttp://www.pubmedcentral.nih.gov/articlerender.fcgi?artid=PMC4170722

10. Burgess S, Timpson NJ, Ebrahim S, Smith GD. Mendelian randomization: Where are we now and where are we going? *Int J Epidemiol*. 2015;**44**(2):379–388.

11. Burgess S, Bowden J. Integrating summarized data from multiple genetic variants in Mendelian randomization: bias and coverage properties of inverse-variance weighted methods. 2015; Available from: http://arxiv.org/abs/1512.04486

12. Burgess S, Bowden J, Fall T, Ingelsson E, Thompson SG. Sensitivity analyses for robust causal inference from Mendelian randomization analyses with multiple genetic variants. *Epidemiology* [Internet]. 2016;**204**:1. Available from: http://content.wkhealth.com/linkback/openurl?sid=WKPTLP:landingpage&an=00001648-900000000-98953

13. Bowden J, Greco M F Del, Minelli C, Davey Smith G, Sheehan N, Thompson J. A framework for the investigation of pleiotropy in two-sample summary data Mendelian randomization. *Stat Med* [Internet]. Wiley-Blackwell; 2017 [cited 2018 Dec 13];**36**(11):1783–1802. Available from: http://www.ncbi.nlm.nih.gov/pubmed/28114746

14. Bowden J, Davey Smith G, Haycock PC, Burgess S. Consistent Estimation in Mendelian Randomization with Some Invalid Instruments Using a Weighted Median Estimator. *Genet Epidemiol*. 2016;**40**(4):304–314.

15. Burgess S, Bowden J, Dudbridge F, Thompson SG. Robust instrumental variable methods using multiple candidate instruments with application to Mendelian randomization. 2016;1–38. Available from: http://arxiv.org/abs/1606.03729

16. Staley JR, Burgess S. Semiparametric methods for estimation of a nonlinear exposure-outcome relationship using instrumental variables with application to Mendelian randomization. *Genet Epidemiol* [Internet]. 2017 May 1 [cited 2017 Jul 6];**41**(4):341–352. Available from: http://doi.wiley.com/10.1002/gepi.22041
