# Supplementary material for: The relationship between sleep duration, cognition and dementia: a Mendelian randomization study
Source: Int J Epidemiol. 2019 May 7;48(3):849–60. doi: 10.1093/ije/dyz071 (PMC6659373; doi:10.1093/ije/dyz071)
Supplement: dyz071_Supplementary_Materials [file dyz071_supplementary_materials.zip › dyz071-suppl_data/Supplementary Figures.docx]

**SUPPLEMENTARY FIGURES**

**
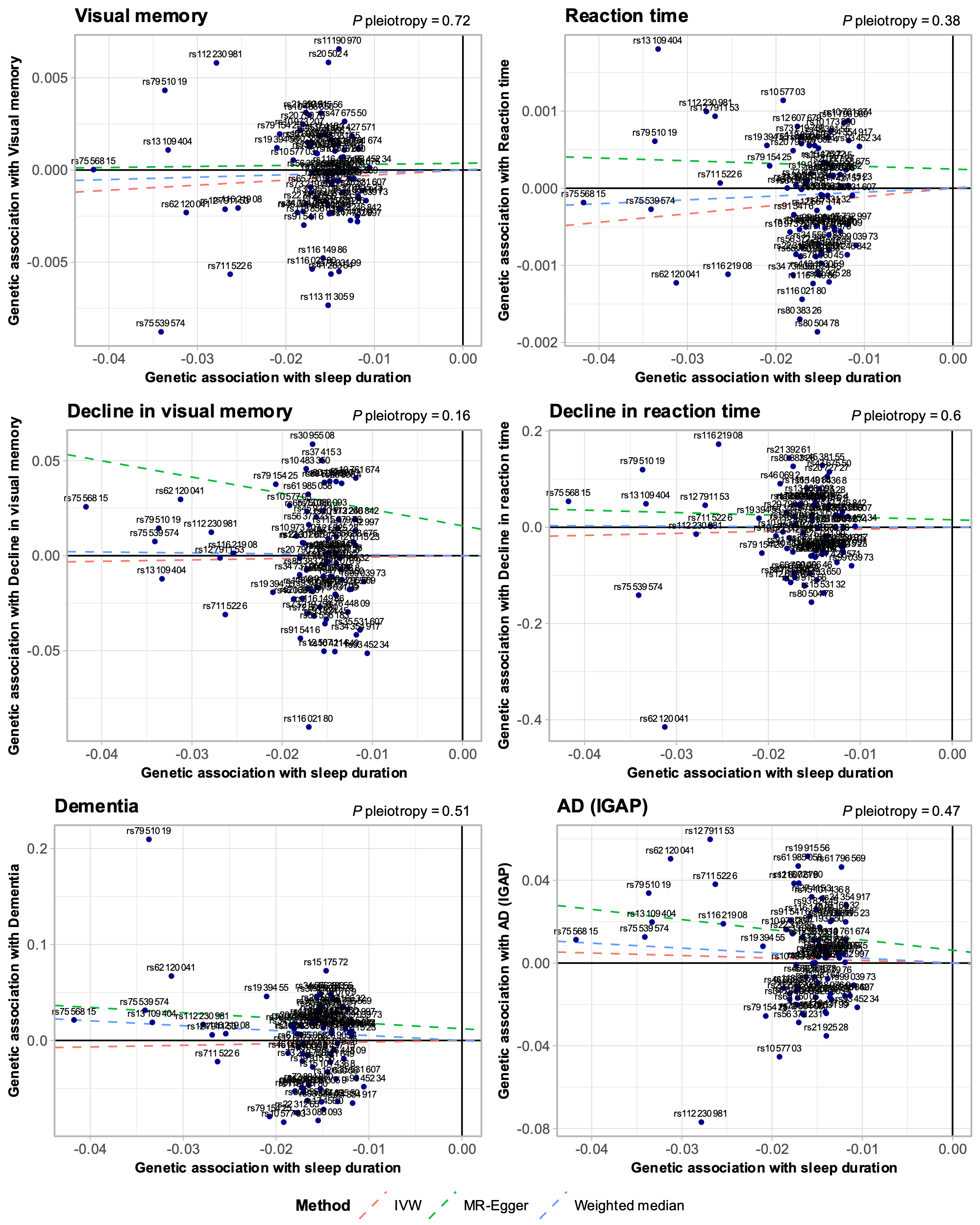
**

**SFigure 1.** Genetic associations with exposure and outcomes; Numbers represent individual single-nucleotide polymorphism (SNP) regression coefficient from simple linear / logistic regression of the outcome on a SNP. *P* Pleiotropy = *P*-value for overall horizontal pleiotropic effect as indicated by intercept from MR-Egger regression; IVW = inverse-variance weigthed; MR-Egger = Mendelian randomization – Egger regression; AD = Alzheimer’s disease; IGAP = International Genomics of Alzheimer’s Disease Project.

**
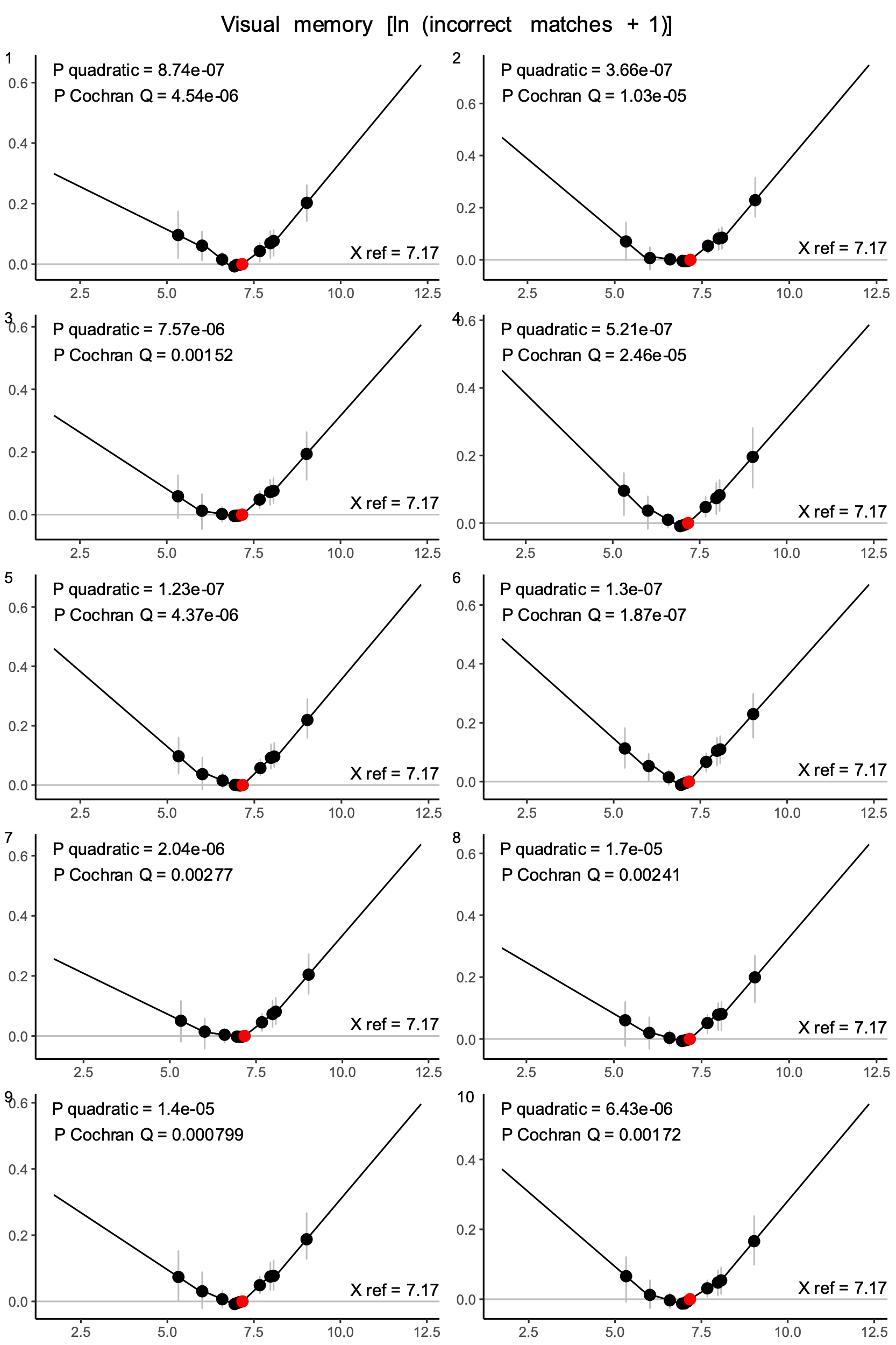
**

**SFigure 2A.** Non-linear MR results from piecewise linear model with 10 strata, estimated from de-discretised sleep duration variable (adding small random variability through Monte Carlo simulation); Each plot represents different simulation (up to 10); Black dots [gray vertical lines] = *localized average causal effect* [95% confidence interval] in each stratum; red dots = mean sleep duration used as reference point (X ref); *P* quadratic / Cochran Q = *P*-value for non-linearity from quadratic / Cochran Q test; Log (incorrect matches + 1) = natural-log of [number of incorrect matches (errors made) in visual memory test + 1]

**
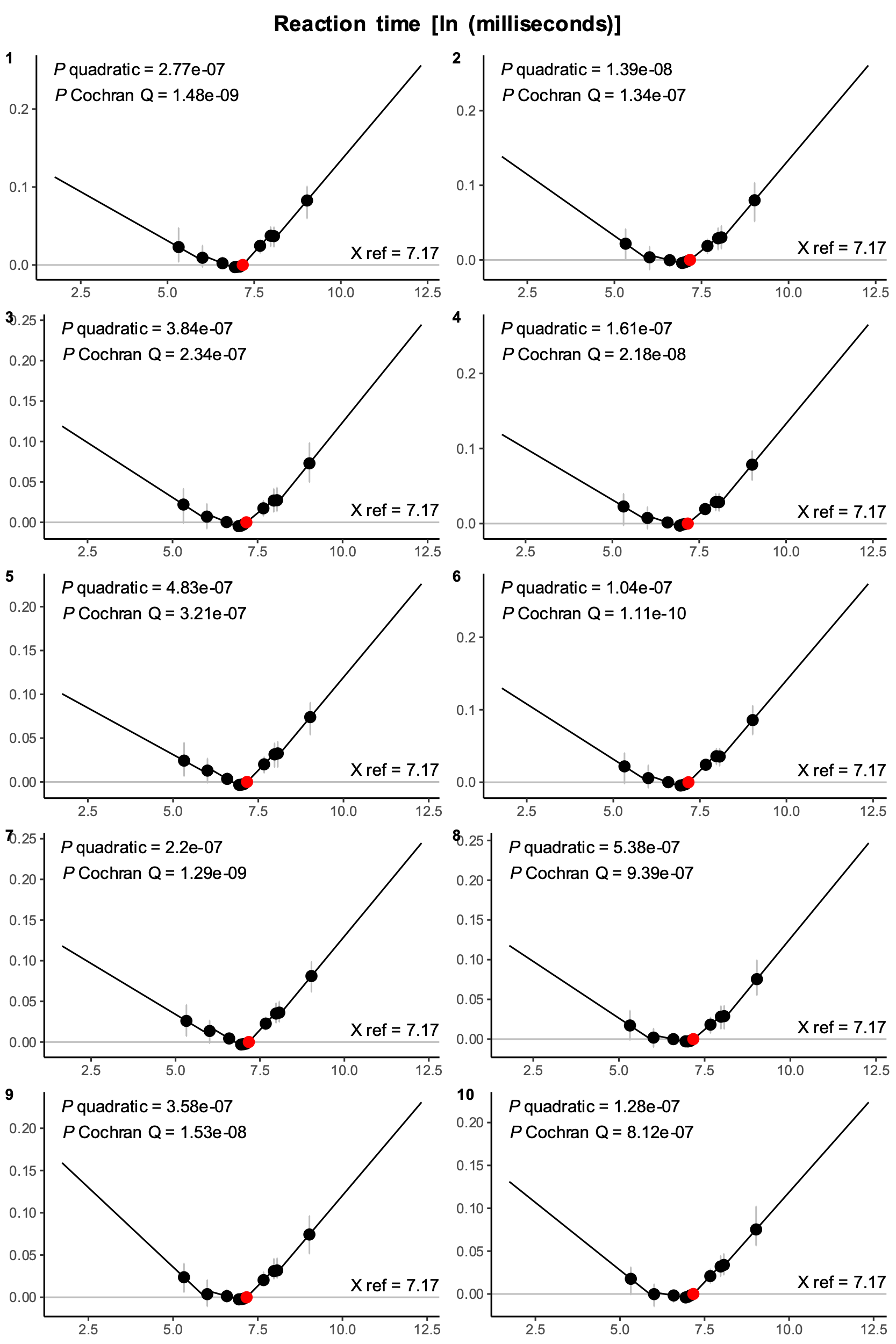
**

**SFigure 2B.** Non-linear MR results from piecewise linear model with 10 strata, estimated from de-discretised sleep duration variable (adding small random variability through Monte Carlo simulation); Each plot represents different simulation (up to 10); Black dots [gray vertical lines] = *localized average causal effect* [95% confidence interval] in each stratum; red dots = mean sleep duration used as reference point (X ref); *P* quadratic / Cochran Q = *P*-value for non-linearity from quadratic / Cochran Q test; Log (milliseconds) = natural-log milliseconds of reaction time.
